# Social determinants of abdominal tuberculosis severity: An experience from a large tertiary care center

**DOI:** 10.12669/pjms.42.8.15840

**Published:** 2026-08

**Authors:** Abdul Samad, Ali Akbar, Nazish Butt, Omar Idris

**Affiliations:** 1 Abdul Samad, Department of Gastroenterology, Jinnah Postgraduate Medical Centre, Karachi, Pakistan; 2 Ali Akbar, Department of Gastroenterology, Jinnah Postgraduate Medical Centre, Karachi, Pakistan; 3 Nazish Butt, Department of Gastroenterology, Jinnah Postgraduate Medical Centre, Karachi, Pakistan; 4 Omar Idris, Department of Gastroenterology, Jinnah Postgraduate Medical Centre, Karachi, Pakistan

**Keywords:** Abdominal tuberculosis, Intestinal tuberculosis, Malnutrition, Socioeconomic status, Social determinants

## Abstract

**Background and Objective:**

Abdominal tuberculosis is a concern in developing countries due to socioeconomic factors, influencing disease severity. This study at Jinnah Postgraduate Medical Centre (JPMC), Karachi, aimed to explore the link between social determinants like socioeconomic status, residence, co-morbidities, addiction patterns, and abdominal tuberculosis severity in patients.

**Methodology:**

A descriptive cross-sectional study was conducted at the Department of Gastroenterology, JPMC, Karachi. Study duration was 16 months from September 26, 2024 to January 26, 2026. A total of 503 patients diagnosed with intestinal tuberculosis were included. Data on social determinants such as socioeconomic status, residence, addiction, HIV status, diabetes, and malnutrition were collected. Disease severity was categorized as mild, moderate or severe based on clinical and radiological, endoscopic assessment. Associations between social determinants and abdominal tuberculosis severity were analyzed using Chi-square tests with a significance level of p ≤ 0.05.

**Results:**

The sample was nearly evenly split by gender and had a mean age of 47.7 years. Most participants came from urban areas and low to middle socio-economic backgrounds. Notably, 20.3% were HIV-positive, 4.2% had diabetes, and nearly one-third (32.7%) were malnourished. Addictive behaviors were common, with 27.8% chewing tobacco and 15% smoking. The severity of intestinal TB was classified as mild (39.9%), moderate (30.7%), or severe (29.4%), based on predefined criteria. While gender, socio-economic status, residence, and HIV status were not significantly associated with disease severity, diabetes (*p* = 0.029), malnutrition (*p* = 0.001), type of addiction (*p* = 0.001), and geographic location (*p* = 0.001) showed significant correlations.

**Conclusion:**

Abdominal tuberculosis severity is significantly influenced by factors like diabetes, malnutrition, type of addiction, and geographic location but not with socioeconomic status, gender, and residence.

## INTRODUCTION

Abdominal tuberculosis, constitutes 1-3% of all tuberculosis cases and 5% of non-pulmonary TB cases worldwide.^1^ Whereas, Pakistan ranks fifth among high-burden TB countries globally. In our country sees around 510,000 new TB cases annually, with 15,000 developing drug-resistant TB yearly, forming 61% of the WHO Eastern Mediterranean Region’s TB burden.^2^ In Pakistan, it is the most prevalent form of extra-pulmonary TB.^3^

In developing nations, factors like sputum ingestion, hematogenous spread, or cow milk contamination can lead to intestinal tuberculosis, mainly affecting the terminal ileum and cecum. Symptoms include abdominal pain, swelling, obstruction, hematochezia, mass, fever, weight loss, and night sweats. Distinguishing between Crohn’s disease and intestinal tuberculosis is often difficult due to similar features, especially in the absence of clear tuberculosis risk factors in endemic regions. Treatment for intestinal tuberculosis typically mirrors that for pulmonary TB.^4^

Socio-economic determinants of health, shape individuals from birth to aging. These factors, beyond medical care, greatly influence health and tuberculosis epidemiology by impacting risk exposure, disease progression, diagnosis time, treatment compliance, and outcomes.^4^-^5^ Exposure to Mycobacterium tuberculosis (MTB) is affected by social behavior, overcrowding, and poor ventilation conditions. Delayed diagnosis, malnutrition, and economic barriers worsen disease susceptibility. TB stigma, tied to lack of support, can hinder treatment compliance and outcomes.^6^

In recent years, the study of TB risk factors and social determinants has intensified. Key factors such as HIV, smoking, diabetes, alcohol use, under-nutrition, overcrowding, poor housing, and economic hardship play a significant role. Areas with high TB incidence often coincide with high rates of HIV, incarceration, overcrowding, unemployment, and immigration.^7^,^8^ In a study, an analysis that included the 22 High TB Burden Countries estimated the population attributable fraction of malnutrition (27%), smoking (23%), HIV (19%), diabetes (6%) and alcohol abuse (13%).^9^

Impact of social factors on tuberculosis is well known, but its influence on severity of abdominal tuberculosis is less understood. ATB often present with non-specific symptoms leading to a diagnostic delays, and advanced disease and complications at diagnosis, prolonged hospital stay and healthcare cost. Knowledge of this association will help to identify at- risk population with poor outcome. This will lead to formulation of strategic interventions in high burden areas. By highlighting social factors affecting ATB severity, the study can guide targeted policies and allocation of resources to reduce morbidity and mortality in timely fashion.

## METHODOLOGY

The study was conducted at the Department of Gastroenterology, Jinnah Postgraduate Medical Centre (JPMC), Karachi. Study duration was 16 months from September 26, 2024 to January 26, 2026 following the approval from institutional review board. It employed a descriptive cross-sectional design to assess the social determinants of Abdominal tuberculosis in patients presenting to a large tertiary care hospital. A non-probability consecutive sampling technique was used to recruit a total of 503 patients.

The inclusion criteria for the study encompassed patients diagnosed with intestinal tuberculosis as per operational definitions, individuals above the age of 14 years, and both male and female participants. Patients diagnosed with solid organ malignancies, hematological malignancies, those who had undergone organ transplantation, were excluded from the study.

### Ethical Approval:

After obtaining approval from the hospital’s Ethical Committee (NO.F.2-81/2024-GENL/82/JPMC dated: September 26, 2024) and securing written informed consent from all participants, eligible patients meeting the inclusion criteria were enrolled in the study.

The selected patients were assessed for social determinants of intestinal tuberculosis, which included socioeconomic status by modified Kuppuswamy scale,^10^ place of residence, co-infections such as HIV, substance addiction, and comorbidities like diabetes mellitus (DM), chronic liver disease (CLD), and chronic kidney disease (CKD).

Data were collected using a structured proforma designed to document relevant demographic and clinical information. The assessment of intestinal tuberculosis was based on ultrasonographic or CT Scan abdomen findings of Ascites, Peritoneal thickening, abdominal lymphadenopathy, bowel wall thickening, and solid organ involvement. Colonoscopy findings consistent with ulcers, polypoidal lesions, strictures, deformed lumen, or ileo-cecal valve. Mucosal biopsies showing granuloma formation, Acid Fast Bacilli and positive Gene Xpert on tissue samples and sputum examination for microscopy. Ascitic fluid was sent for ADA levels in patients with ascites. Exclusion criteria were used to minimize potential confounders.^11^

### Operational definitions of diseases severity:

As there is no universally accepted scoring system or criteria for defining Abdominal TB severity, we operationally defined severity on the basis of clinical, radiological, endoscopic findings and presence of complications.

### Mild disease:

Was defined as presence of localized intestine involvement on imaging with mild clinical symptoms of abdominal pain and weight loss in the absence of obstruction, bleeding perforation and complications.

### Moderate disease:

Multisegmental intestinal involvement along with ascites and lymphadenopathy with moderate symptoms severity, without evidence of perforation, bleeding, obstruction or complications.

### Severe disease:

Abdominal tuberculosis which presents with severe symptoms of obstruction, perforation, bleeding, which requires intervention or surgery.

### Statistical analysis:

Was performed using SPSS version 25.0. Mean and standard deviation were calculated for quantitative variables such as age, while categorical variables, including gender, socioeconomic, residence, smoking status, HIV status, substance use, diabetes, and malnutrition, were analyzed using frequency and percentage distributions. The chi-square test was applied to assess associations between social determinants and abdominal tuberculosis severity, with a p-value of ≤0.05 considered statistically significant. Furthermore, the magnitude and precision of observed differences were reported by effect sizes with 95 percent confidence intervals, in order to ensure that the difference is not only statistically significant but also clinically relevant.

## RESULTS

The distribution of demographic, socioeconomic, health, and geographic characteristics of the 503 participant is given in Tale-I. The sample included slightly more males (51.1%) than females (48.9%), with a mean age of 47.7 years (±20.7). Regarding socio-economic status, 41.6% of participants were in the low-income group, 35.0% in the middle, and 23.4% in the high-income group. A majority resided in urban areas (57.1%), while 42.9% were from rural areas.

**Table-I:** Frequency distribution of different variables (n=503).

Variables		Frequency	Percent	95% CI (%)
Gender	Male	257	51.1%	46.7–55.5
	Female	246	48.9%	44.5–53.3
Age (years)	47.72±20.79			45.90–49.54 years
Socio-economic status	Low	209	41.6%	37.3–45.9
	Middle	176	35.0%	30.8–39.2
	High	118	23.5%	19.8–27.2
Residence	Rural	216	42.9%	38.6–47.2
	Urban	287	57.1%	52.8–61.4
HIV status	Yes	103	20.5%	17.0–24.0
	No	400	79.5%	76.0–83.0
Diabetes	Yes	21	4.2%	2.4–5.9
	No	482	95.8%	94.1–97.6
Malnutrition	Yes	163	32.4%	28.3–36.5
	No	340	67.6%	63.5–71.7
Addiction	None	171	34.0%	29.9–38.1
	Alcohol	69	13.7%	10.7–16.7
	IV drug abuser	13	2.6%	1.2–4.0
	Smoker	77	15.3%	12.2–18.4
	Inhalational substance abuse	33	6.6%	4.4–8.7
	Tobacco chew	140	27.8%	23.9–31.7
Division of Sindh	Hyderabad	77	15.3%	12.2–18.4
	Karachi	182	36.2%	32.0–40.4
	Larkana	48	9.5%	7.0–12.1
	Shaheed Benazirabad	130	25.8%	22.0–29.7
	Sukkur	66	13.1%	10.2–16.1
Districts of Karachi	Central	26	12.7%	9.2–19.4
	East	39	24.3%	15.4–27.4
	West	36	21.6%	14.0–25.6
	North	33	18.9%	12.5–23.7
	South	24	10.8%	8.3–18.1
	Kemari	24	11.7%	8.3–18.1
Severity of disease	Mild	201	40.0%	35.7–44.3
	Moderate	154	30.6%	26.6–34.6
	Severe	148	29.4%	25.4–33.4

Among the participants, 20.5% were HIV positive, 4.2% had diabetes, and 32.4% were malnourished. In terms of addiction, 34.0% had no addiction history, while others reported using tobacco (27.8%), alcohol (13.7%), or smoking (15.3%), with a small proportion being IV drug users (2.6%) or Inhalational substance abuse users (6.6%).

Geographically, most participants were from Karachi (36.2%) and Shaheed Benazirabad (25.8%), followed by Hyderabad (15.7%), Sukkur (14.4%), and Larkana (7.8%). Within Karachi, East (24.3%) and West (21.6%) districts had the highest representation. The mean ADA level, was 53.25 (±26.56). Based on predefined categories 39.9% of patients had mild disease, 30.7% moderate, and 29.4% severe.

The relationship between various social determinants and the severity of intestinal tuberculosis is shown in Table-II. Gender, socio-economic status, residence, and HIV status were not significantly associated with disease severity (p > 0.05). However, certain factors showed significant associations.

**Table-II:** Association between social determinants and intestinal tuberculosis severity.

Variables		Severity of disease			p-value
		Mild	Moderate	Severe	
Gender	Male	105(42.7%)	69(28.0%)	72(29.3%)	0.380
	Female	96(37.4%)	85(33.1%)	76(29.6%)	
Socio-economic status	Low	79(37.8%)	58(27.8%)	72(34.4%)	0.068
	Middle	84(47.7%)	50(28.4%)	42(23.9%)	
	High	38(32.2%)	46(39.0%)	34(28.8%)	
Residence	Rural	85(39.4%)	64(29.6%)	67(31.0%)	0.786
	Urban	116(40.4%)	90(31.4%)	81(28.2%)	
HIV status	Yes	49(47.6%)	36(35.0%)	18(17.5%)	0.061
	No	152(38.0%)	118(29.5%)	130(32.5%)	
Diabetes	Yes	3(14.3%)	5(23.8%)	13(61.9%)	0.003
	No	198(41.1%)	149(30.9%)	135(28.0%)	
Malnutrition	Yes	85(52.1%)	29(17.8%)	49(30.1%)	0.001
	No	116(34.1%)	125(36.8%)	99(29.1%)	
Addiction	None	87(50.9%)	31(18.1%)	53(31.0%)	0.001
	Alcohol	34(49.3%)	16(23.2%)	19(27.5%)	
	IV drug abuser	5(38.5%)	8(61.5%)	0(0.0%)	
	Smoker	25(32.5%)	20(26.0%)	32(41.6%)	
	Inhalational substance abuse	15(45.5%)	8(24.2%)	10(30.3%)	
	Tobacco chew	35(25.0%)	71(50.7%)	34(24.3%)	
Division of Sindh	Hyderabad	39(50.6%)	17(22.1%)	21(27.3%)	0.001
	Karachi	79(43.4%)	54(29.7%)	49(26.9%)	
	Larkana	22(45.8%)	6(12.5%)	20(41.7%)	
	Shaheed Benazirabad	25(19.2%)	66(50.8%)	39(30.0%)	
	Sukkur	36(54.5%)	11(16.7%)	19(28.8%)	

Patients with diabetes had a notably higher proportion of severe disease (61.5%, *p* = 0.003). Malnutrition was also associated with severity, with 52% of malnourished individuals having mild TB but 30% presenting with severe disease (*p* = 0.001). Addiction type also had a significant correlation (*p* = 0.001); for instance, smokers and tobacco chewers showed higher proportions of severe and moderate disease, respectively.

There was a significant geographic variation in disease severity (*p* = 0.001). Participants from Shaheed Benazirabad had the highest rate of moderate disease (50.6%), while severe disease was more common in Larkana (41.7%) and Sukkur (29.5%).

## DISCUSSION

Our study examined the association between social determinants and abdominal tuberculosis severity among patients at a large tertiary care hospital in Karachi, Pakistan. We found malnutrition, diabetes mellitus and substance abuse are significantly associated with severity of the disease.

Our study found that 41.5% of abdominal tuberculosis patients belonged to the low-income group, while 57.2% resided in urban areas. These findings align with global studies indicating that poverty, overcrowding, and poor sanitation contribute to tuberculosis transmission and progression.^12^ A study in India by Agarwal also reported a higher prevalence of TB among lower socioeconomic classes, where malnutrition and delayed healthcare access exacerbate disease severity.^13^ However, our findings did not show a significant difference in abdominal tuberculosis severity across socio-economic groups (p = 0.141). These results contradict the study that suggests poverty increases the disease risk.^14^

HIV co-infection is a known risk factor for extrapulmonary tuberculosis, including abdominal TB, due to the associated immunosuppression.^15^ In our study, 20.3% of abdominal tuberculosis patients were HIV-positive, which is comparable to regional data showing HIV-TB co-infection rates between 15-25% in endemic areas.^16^ However, HIV status was not significantly associated with abdominal tuberculosis severity (p = 0.078), which contradicts previous studies suggesting that HIV-positive TB patients tend to have more severe disease and complications.^17^ This discrepancy may be due to the use of antiretroviral therapy (ART) in our study population, which mitigates TB severity in HIV-infected individuals.

Diabetes is a known risk factor for increased TB severity due to impaired immune responses.^18^ Our study observed that diabetic patients had a higher proportion of severe abdominal tuberculosis cases (61.5%), this was statistically significant (p = 0.029). Studies from Southeast Asia and Sub-Saharan Africa have demonstrated that TB patients with diabetes have a 2-3 times higher risk of developing severe disease and complications.^19^

Malnutrition has been widely recognized as both a cause and consequence of tuberculosis.^20^ Our study found that 32.7% of abdominal tuberculosis patients were malnourished, and malnutrition showed a significant association with severe abdominal tuberculosis (p = 0.001). A meta-analysis of TB and malnutrition found that undernourished patients have a higher bacterial burden, slower response to treatment, and higher mortality rates.^21^ In a study conducted in Bangladesh, malnourished TB patients had lower treatment success rates and higher rates of unfavorable outcomes. Whereas our study has not focused on interventions, but it highlights the potential significance of nutritional support in high risk groups.^22^

Substance abuse, particularly tobacco and alcohol consumption, is a recognized risk factor for TB transmission, disease progression, and poor treatment outcomes.^23^ In our study, 27.8% of patients chewed tobacco, 15.0% were smokers, and 13.7% consumed alcohol, with a higher prevalence of moderate-to-severe disease in smokers (p = 0.001). A systematic review by Slama et al. found that tobacco smokers have twice the risk of severe TB and treatment failure.^24^ Similarly, alcohol use disorder has been linked to increased TB mortality due to its immunosuppressive effects.^25^ Although causality could not be inferred, our study suggests the need for targeted smoking and alcohol cessation and behavioral therapies programs for abdominal tuberculosis patients.

Geographical variations in abdominal tuberculosis prevalence and severity are influenced by healthcare access, socio-economic disparities, and endemic infection rates. In our study, the highest number of cases were reported from Karachi (36.3%), followed by Shaheed Benazirabad (25.8%), Sukkur (14.4%), and Hyderabad (15.7%). Regional differences in abdominal tuberculosis severity were statistically significant (p = 0.001). Previous studies have reported higher TB prevalence in rural and underdeveloped regions of Pakistan, where limited healthcare access and poor living conditions contribute to disease burden.^2^

### Limitations:

Our study design was cross-sectional, preventing us from establishing causality between social determinants and abdominal tuberculosis severity.

## CONCLUSION

Abdominal tuberculosis severity is significantly influenced by factors like diabetes, malnutrition, type of addiction, and geographic location but not with socioeconomic status, gender, and residence.

### Recommendations:

Future studies with larger cohorts and longitudinal designs are needed to confirm these findings.

### Author’s Contribution:

**AS:** Conceived, designed the study. Literature search.

**AA:** Interpretation, analysis of data and critical review of the manuscript.

**NB:** Designed the study, critical review and responsible for the integrity of the study.

**OI:** Written and edited the manuscript, reviewed and final approval of manuscript.
